# Dynamic Exploration of Resting-State Brain Attractors Altered in Major Depressive Disorder

**DOI:** 10.3390/e28020191

**Published:** 2026-02-09

**Authors:** Leonor Abreu, Joana Cabral

**Affiliations:** 1 Institute for Systems and Robotics (ISR-Lisboa), Instituto Superior Técnico, University of Lisbon, 1049-001 Lisboa, Portugal; leonoabreu@gmail.com; 2 Department of Bioengineering, Instituto Superior Técnico, University of Lisbon, 1049-001 Lisboa, Portugal

**Keywords:** major depressive disorder, LEiDA, resting-state networks, brain dynamics

## Abstract

Major depressive disorder (MDD) represents a heterogeneous condition lacking reliable neurobiological biomarkers and a mechanistic understanding. Time-resolved characterization of brain dynamics reveals that mental health is associated with a characteristic dynamical regime, exhibiting spontaneous switching between a repertoire of ghost attractor states forming resting-state networks. Analysing resting-state fMRI data from 848 patients with MDD and 794 healthy controls across 17 sites in China (REST-meta-MDD) using Leading Eigenvector Dynamics Analysis (LEiDA), we found patients with MDD exhibited significantly reduced default mode network (DMN) occupancy (*p* < 0.001; Hedges’ *g* = −0.51) and increased occipito–parieto–temporal state occupancy (*p* < 0.001; Hedges’ *g* = 0.42), suggesting compensatory dynamical rebalancing. These findings extend prior observations of DMN disruption in MDD, aligning with the emerging dynamical systems framework for mental health to advance the mechanistic understanding of MDD pathophysiology.

## 1. Introduction

Major depressive disorder (MDD) is a heterogeneous psychiatric condition characterized by mood disturbances, anhedonia, cognitive impairments, and neurovegetative symptoms [1]. MDD affects over 330 million individuals worldwide and ranks as a leading cause of disability and mortality, with a significant socioeconomic burden [2,3]. Despite its prevalence, MDD lacks reliable mechanistic biomarkers and personalized treatment stratification, due in part to clinical and genetic heterogeneity [4,5].

Recent advances in resting-state functional MRI (rs-fMRI) offer promising non-invasive approaches for characterizing intrinsic brain network dysfunction in MDD. Rs-fMRI captures whole-brain connectivity patterns without task demands, enabling scalable biomarker development across diverse populations and sites [6,7]. It has revealed abnormalities in key brain networks, such as the default mode network (DMN), frontoparietal control network, and salience network, linked to core MDD symptoms including rumination and executive dysfunction [8,9].

Dynamic functional connectivity (dFC) approaches, including Leading Eigenvector Dynamics Analysis (LEiDA), capture temporal fluctuations in network states at fine timescales, providing mechanistic insights beyond static connectivity measures [9,10]. Prior large-scale analyses, notably from the REST-meta-MDD consortium, have demonstrated heterogeneous DMN dysfunction, altered inter-network coupling, and clinical correlates across thousands of patients [7]. However, technical variability in preprocessing and analytic methods challenges the reproducibility of findings [11].

This secondary data analysis leverages REST-meta-MDD rs-fMRI data to investigate alterations in the temporal dynamics of functional networks in MDD pathophysiology without any particular network or region of interest [10]. Using LEiDA applied to AAL-90 parcellated time series and k-means clustering across multiple resolutions, the present study assesses group differences between patients with MDD and healthy controls (HC) in the fractional occupancy (FO) of recurrent attractor states detected in brain dynamics, revealing significant alterations in the dynamical landscape of brain activity in MDD compared with controls.

## 2. Materials and Methods

### 2.1. Study Design

The analysis focused exclusively on between-group comparisons of dFC metrics, without incorporating clinical covariates such as symptom severity, depression subtypes, or behavioral measures. This restriction ensured methodological uniformity, given the heterogeneity and incomplete availability of detailed clinical variables across the multisite dataset [7].

Detailed clinical covariates (e.g., symptom severity, illness duration, episode status, and depression subtypes) were not modelled in the present analysis, as these measures were heterogeneous and incompletely available across REST-meta-MDD cohorts, and the preregistered analytic plan focused on robust group-level differences in dFC metrics. This choice prioritized sample size and methodological uniformity over fine-grained clinical stratification.

### 2.2. Quality Control and Sample Selection

The initial secondary data analytic plan included rs-fMRI data from 1300 patients with MDD and 1128 healthy controls recruited across 25 independent cohorts in China [7]. Rigorous quality control (QC) procedures identified significant data integrity challenges—such as spatial normalization errors, incomplete brain coverage, and motion artifacts—even at high-scoring sites. Consequently, the analysis was limited to a strictly quality-assured subsample, adhering to REST-meta-MDD criteria to ensure methodological validity and reproducibility, as previously performed by Yan and colleagues [7]. In more detail, QC inspection was performed through a multi-step protocol combining automated metrics and manual review [7]. Spatial normalization accuracy was verified through visual inspection of warped images against the Montreal Neurological Institute (MNI) template [7]. Subjects exhibiting normalization errors, incomplete brain field-of-view coverage, or excessive head motion exceeding a framewise displacement threshold of 0.2 mm were excluded [12]. Furthermore, cohorts with fewer than ten subjects per group were omitted to maintain statistical robustness [7]. These rigorous criteria resulted in a final quality-assured sample of 848 individuals with MDD and 794 HC from 17 sites (freely available for download at rfmri.org/REST-meta-MDD), following REST-meta-MDD consortium criteria to maximize data reliability and analytic validity [7].

No additional statistical harmonisation across sites (e.g., ComBat or site-specific scaling) was applied, and the site was not included as a covariate in the permutation tests. Given the large number of centres and unbalanced sample sizes, the design was optimized to detect robust overall group differences rather than site-specific estimates [7].

### 2.3. Participants

Inclusion required a clinical diagnosis of MDD based on structured clinical interviews adhering to either ICD-10 or DSM-IV criteria [7]. Healthy controls were rigorously screened to exclude psychiatric and neurological disorders [7]. Participant demographic data were harmonised across sites, with age inclusion restricted to 18–65 years [7]. The final sample included 67% females in the MDD group (mean age 36.2 years, SD = 12.1) and 65% females in controls (mean age 35.7 years, SD = 11.9) [7]. Education levels were standardized to comparable categories, with the majority completing secondary education or higher [7].

Clinical profiles of the MDD group comprised an average illness duration of 4.2 years (SD = 5.1), with 52% in their first episode and 48% classified as recurrent [7]. Medication status included 60% medicated and 40% unmedicated participants [7]. Symptom severity was primarily quantified using the Hamilton Depression Rating Scale (HDRS-17), with a mean score of 22.7 (SD = 5.4) [7]. Other clinical scales were variably collected across cohorts but were not analysed here due to inconsistent availability [7]. Medication status was not incorporated as a covariate or stratification factor in the current group-level comparisons, in line with the secondary analysis plan of REST-meta-MDD.

### 2.4. Imaging Acquisition

Rs-fMRI data from the REST-meta-MDD consortium were acquired predominantly on 3T MRI scanners across all sites, with approximately 10% of scans performed on 1.5T systems [7]. Harmonised imaging protocols were implemented, adhering to the consortium standardized sequence parameters: echo-planar imaging (EPI) sequence, repetition time (TR) ~2000 ms, echo time (TE) ~30 ms, flip angle 90°, field of view 220 × 220 mm, matrix size 64 × 64, slice thickness 3–4 mm, and scan length ranging between 180 and 240 volumes [7]. Participants were uniformly instructed to remain awake and relaxed with eyes closed during scanning, and data from individuals displaying signs of sleep or excessive motion were excluded [7].

Field strength (1.5T vs. 3T) was not explicitly modelled as a covariate, and no separate analyses by scanner type were conducted. The QC-filtered sample reflects the distribution of field strengths in REST-meta-MDD, which is determined by site-specific recruitment and diagnostic composition [7].

### 2.5. Preprocessing

Rs-fMRI preprocessing employed the DPARSF toolbox [7,13], integrated with SPM12 in MATLAB 2020b [7,14]. The pipeline involved slice-timing correction, head-motion realignment, co-registration of functional and anatomical images, spatial normalization to MNI space using Diffeomorphic Anatomical Registration Through Exponentiated Lie algebra (DARTEL), and smoothing with a 6 mm full-width-at-half-maximum Gaussian kernel [7]. Nuisance regression included the Friston-24 motion parameters (6 motion parameters plus their derivatives and squared terms), white matter, and CSF signals, mitigating motion and physiological noise [15]. Linear trend removal and temporal band-pass filtering (0.01–0.08 Hz) isolated low-frequency fluctuations relevant for resting-state connectivity analyses [7]. Global signal regression was not applied, consistent with REST-meta-MDD standards, to avoid introducing artificial anti-correlations [7].

In this work, we used data parcellated using the anatomically based AAL-90 atlas to align with previous LEiDA studies [16,17] and facilitate anatomical interpretability [17,18].

### 2.6. Data Analysis

Dynamic functional connectivity was estimated using LEiDA, applied to the Hilbert-transformed phase of preprocessed fMRI signals after the band-pass filtering described in Section 2.5 [19]. For LEiDA, parcel-wise time series were further demeaned and detrended, but no additional temporal filtering beyond the 0.01–0.08 Hz band-pass was applied before Hilbert transformation.

At each time point (TR = 2 s), the instantaneous phase-coherence matrix (90 × 90) was decomposed to extract only its leading eigenvector, representing the dominant signal phase organization across the brain [16]. Concatenating eigenvectors across all subjects and time points, k-means clustering was performed using cosine distance for cluster numbers ranging from K = 2 to 20, with 25 replicates per K to ensure stability [16]. Each clustering solution returned K recurrent ‘phase-locking’ states, which were spatially correlated with canonical RSNs [16,17]. Within the LEiDA framework, a ‘phase-locking state’ corresponds to a spatial pattern of phase alignment among signals across all brain areas, detected recurrently over time and across datasets. Each phase-locking state is described by a vector of size 1 × N, where N = 90 is the number of cortical and subcortical brain areas (excluding the cerebellum) in the AAL parcellation scheme [20,21,22]. The values in the vector range between −1 and 1, corresponding to the cosine of the phase difference with respect to the leading eigenvector direction. Specifically, a value of 1 corresponds to zero phase difference (cos(0°) = 1), a value of −1 corresponds to a phase difference of 180 degrees (cos(180°) = −1), and a value of 0 corresponds to a phase difference of 90 degrees (cos(90°) = 0) [20,21,22]. When all values in a phase-locking state are positive, the signals in all brain areas are shifted by less than 90 degrees relative to the leading direction, whereas negative values indicate a shift greater than 90 degrees [20,21,22].

It is important to note that this measure is distinct from the Phase-Locking Value (PLV) commonly used in oscillatory analyses. While PLV quantifies the temporal stability of the phase relationship between a pair of signals, a LEiDA phase-locking state captures a spatial snapshot of instantaneous phase alignment across all brain areas at each time point. A given phase-locking state may occur only briefly, but its recurrent appearance suggests the presence of a ‘ghost attractor’ in dynamical systems terminology—an underlying structure that governs the repeated expression of this particular configuration of phase alignment [20,21,22].

Fractional Occupancy (FO), defined as the proportion of time points assigned to each state, was calculated for each state in each scan. Between-group comparisons used two-sided label-permutation tests with 5000 permutations per state. To control for multiple comparisons across all clustering solutions, we applied a single Bonferroni correction over the total number of tested states (209 across K = 2–20), such that effects were considered statistically significant only if *p* < 0.05/209. This conservative global threshold was used for all inferential claims.

### 2.7. Ethics

This secondary data analysis was conducted in strict accordance with the King’s College London Research Ethics Policy and the ethical standards established by the British Psychological Society (BPS) and the Medical Research Council (MRC), namely the BPS Code of Human Research Ethics and MRC Good Research Practice principles [23,24].

The original data collection adhered to ethical standards approved by the institutional review boards of all participating sites, following the Declaration of Helsinki and the guidelines of the International Committee of Medical Journal Editors (ICMJE).

All individuals provided written informed consent, and the data were fully anonymized prior to analysis. Data transfer and storage complied with the GDPR and institutional data protection policies, utilizing secure, encrypted servers with restricted access at the Universidade do Minho. Investigators submitting analytic plans to the REST-meta-MDD consortium underwent protocol review to ensure compliance with ethical governance and data integrity standards. Adverse events reporting protocols were rigorously followed to maintain participant safety and uphold research integrity throughout.

## 3. Results

### 3.1. Identification of Dynamic Functional Connectivity States

**Figure 1:**
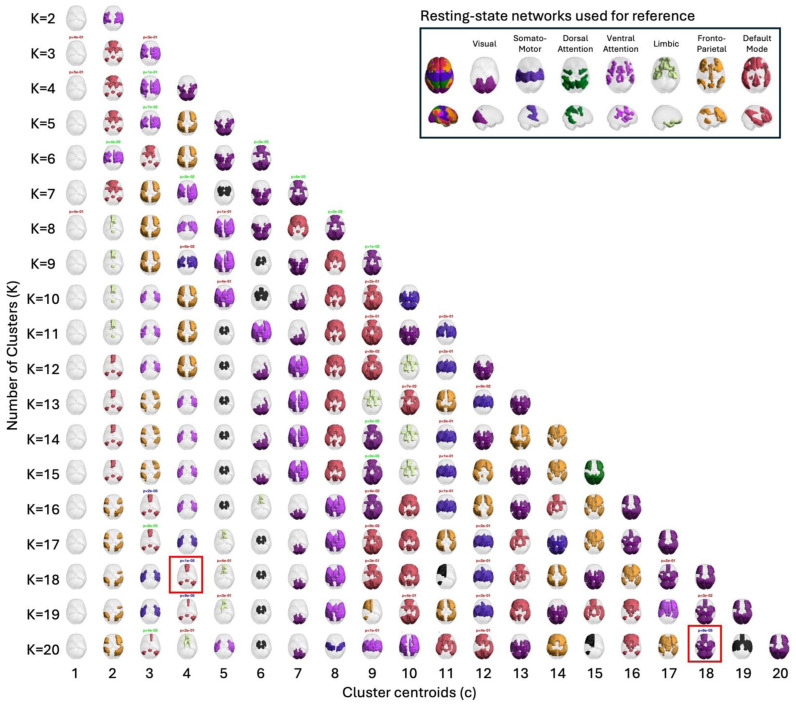
Centroid pyramid of phase-locking (PL) states obtained when clustering the leading eigenvectors into K = 2 to K= 20 clusters. Rows depict clustering solutions at each K; columns display the cluster centroids, sorted from c = 1 to c = K according to decreasing probability of occurrence in all fMRI scans. Each centroid is represented on a transparent brain by rendering the subset of brain regions whose signals align together in phase. The color of the rendering associate serves to associate each cluster centroid with 7 canonical resting state networks shown on to the right hand panel. Centroids colored in black do not overlap with any of these 7 networks. The title above each centroid reports the *p*-value from permutation tests (5000 permutations per state) and the color indicates the level of statistical significance: the most significant in blue for *p* < 0.05/209, green for 0.05/209 ≤ *p* < 0.05/K and red for 0.05/K < *p* < 0.05. Among clusters differing the most between groups (*p* < 0.05/209), those selected for detailed analysis are additionally marked with a red rectangle [17].

In the present study, the use of LEiDA with hierarchical k-means across K = 2–20 was employed to identify and arrange recurring phase-locking (PL) states, as defined above, into a centroid pyramid. This was implemented to visualise continuity and fractionation across resolutions (Figure 1). Rows represent clustering solutions at each K, with clustet centeoids sorted from left to right according to decreasing occupancy in all fMRI scans. Across the range of K explored, most states spatially overlap with canonical resting-state networks such as the Visual, Somatomotor, Dorsal, and Ventral Attention, Limbic, Frontoparietal, and Default Mode networks [17].

Once clustering was performed across a range of K values (2–20), this design entails a large number of statistical tests. To avoid inflating the familywise error rate, all state-level permutation *p*-values were subjected to a single Bonferroni correction across the 209 states, irrespective of K. The two highlighted states (posterior DMN at K = 18; occipito–parieto–temporal at K = 20) are those that survived this global correction, having high chances of being true positives.

### 3.2. Group Differences in State FO

Group differences in FO between MDD and HC were assessed using unpaired, two-sided label-permutation tests (5000 permutations per state). In Figure 1, label colours encode *p*-value significance, with blue marking *p* < 0.05/209 and green marking 0.05/209 ≤ *p* < 0.05/K. Among the blue ones (*p < 0.05/209*), those selected for detailed analysis are additionally marked with a red square. Primary inferences were controlled within each K using Bonferroni. Cross-K summaries are descriptive.

**Figure 2:**
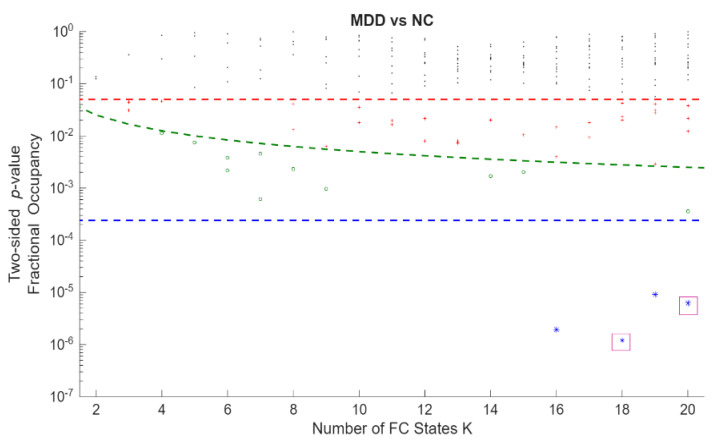
Statistical significance of differences in occupancy between MDD and HC across all Fractional Occupancy (FO states obtained from clustering solutions. The *y*-axis shows state-level *p*-values on a logarithmic scale, and the *x*-axis shows the number of FC states K (2–20). Each point represents the *p*-value of one FC state at a given K. The red horizontal dashed line denotes the nominal significance level *p* = 0.05; the green dashed curve denotes the within-K Bonferroni threshold *p* = 0.05/K, shown as a descriptive reference; and the blue horizontal dashed line denotes the global Bonferroni-corrected threshold *p* = 0.05/209 across all FC states and clustering resolutions, which was the criterion used to define statistical significance in the main analyses (K18C4 (*p* = 1.20 × 10^−6^) and K20C18 (*p* = 6.17 × 10^−6^) were marked with a purple square.

**Figure 3:**
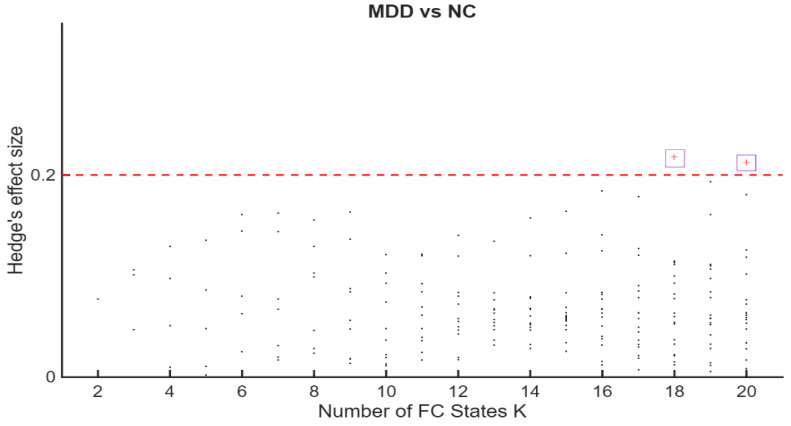
Hedges’ *g* for MDD–HC differences in Fractional Occupancy (FO across K. The horizontal axis indexes K = 2–20. The vertical axis shows Hedges’ ***g***. Positive values indicate higher FO in MDD; negative values indicate higher FO in HC. Dashed reference lines mark conventional benchmarks for small [≈0.2], medium [≈0.5], and large [≈0.8] effect sizes. The highest effect sizes values, corresponding to K = 18 and K = 20 were marked with a purple square.

Permutation *p*-values and effect sizes across K are summarized in Figure 2 and Figure 3, respectively. Effect sizes (Hedges’ *g*) were generally small and centred near zero, with both positive and negative values indicating bidirectional FO differences. Together, these patterns suggest a redistribution of time across states rather than uniform increases or decreases within a single group.

### 3.3. Characterization of Key States

Two states showed statistically significant differences in FO between MDD and HC and were examined in detail: a posterior DMN state at K = 18, cluster 4 (K18C4; *p* = 1.20 × 10^−6^), and an occipito–parieto–temporal state at K = 20, cluster 18 (K20C18; *p* = 6.17 × 10^−6^).

### 3.4. Posterior DMN State (K18C4)

**Figure 4:**
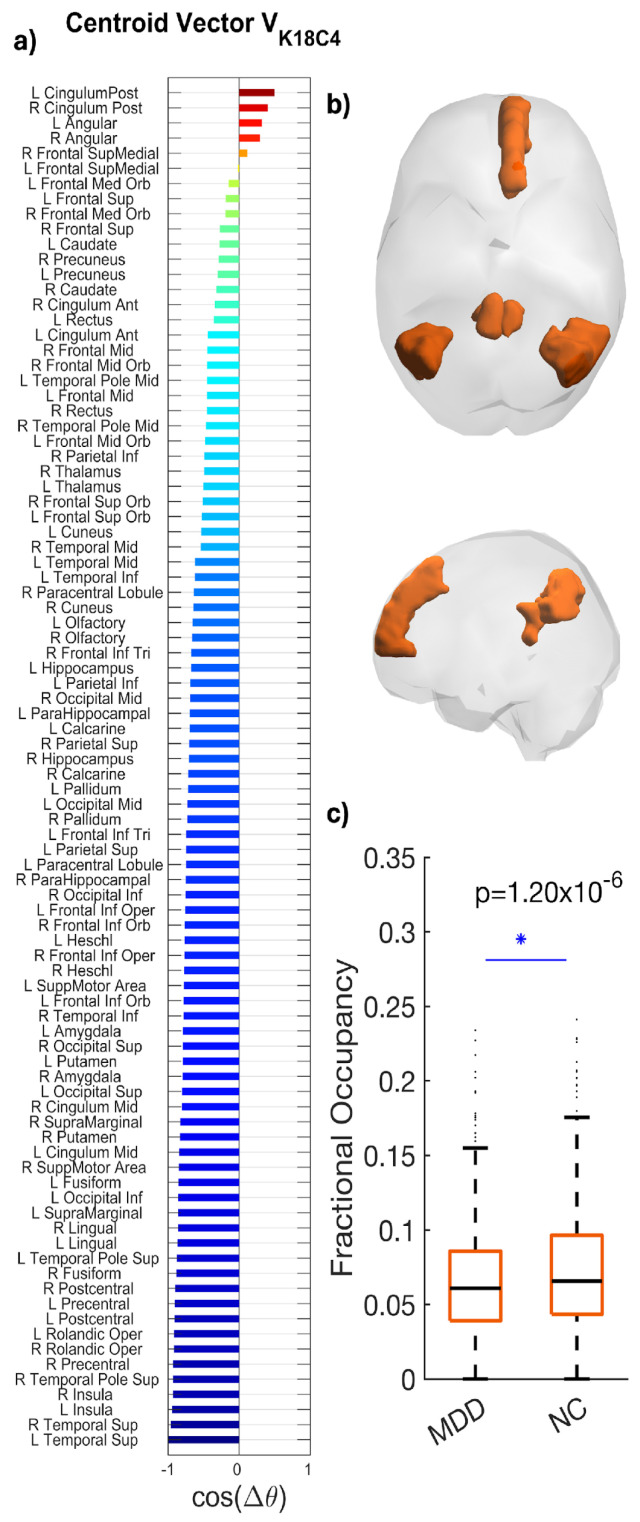
Phase-locking (PL) state occurring significantly less in fMRI scans from patients with MDD compared to controls. (**a**) The N = 90 elements in the centroid vector obtained at K = 18, c = 4 indicate the phase relationships among the N = 90 brain areas with respect to the eigenvector. In more detail, all brain areas with positive values are aligned together in phase. (**b**) LEiDA brain maps display weights with the strongest loadings in the posterior cingulate/precuneus, bilateral angular/inferior parietal, medial prefrontal, and medial/lateral temporal (including hippocampal/parahippocampal) regions. (**c**) Boxplots compare Fractional Occupancy among participants in each group (MDD mean = 0.052; HC mean = 0.071; * *p* = 1.20 × 10^−6^; Hedges’ *g* = 0.218). Boxes depict the interquartile range; horizontal lines mark medians; whiskers indicate variability; points represent outliers among participants in each group. Summary statistics (median and interquartile range) are shown to facilitate distributional interpretation.

K18C4 exhibited a posterior DMN topology, with dominant loading in the posterior cingulate/precuneus and bilateral angular/inferior parietal cortex, extending into medial prefrontal and medial/lateral temporal regions, including hippocampal–parahippocampal areas (Figure 4).

Patients with MDD showed reduced FO compared with HC (MDD: 0.052; HC: 0.071), reflecting an approximately 27% reduction. The between-group difference was statistically significant (*p* = 1.20 × 10^−6^) with a medium effect size (Hedges’ *g* = 0.218; 95% CI [−0.73, −0.29]) (Figure 4). Boxplots generated via LEiDA indicate a downward shift in central tendency for MDD, with comparable dispersion.

### 3.5. State (K20C18)

**Figure 5:**
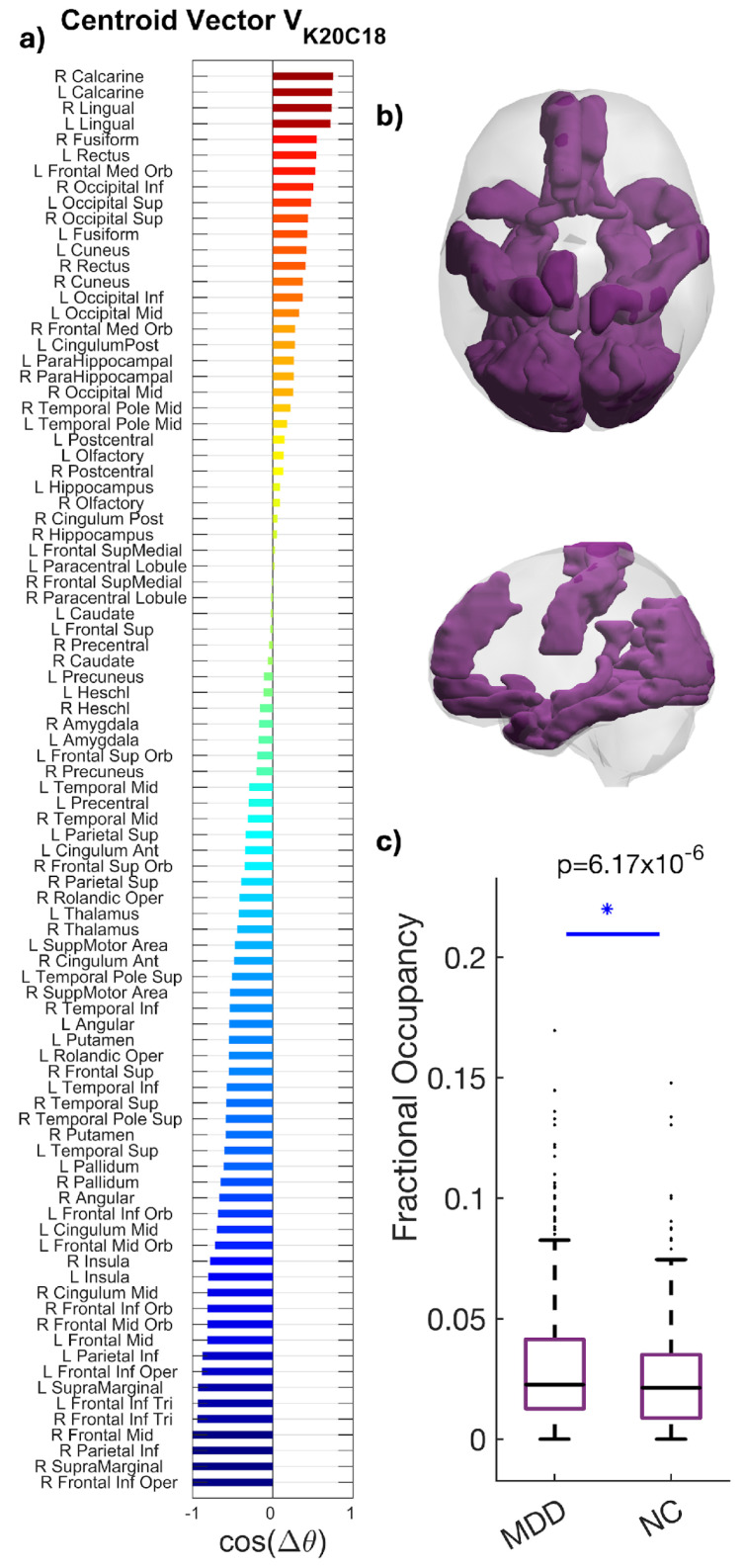
Spatial characterization of the phase-locking (PL) occipito–parieto–temporal state. (**a**) The N = 90 elements in the centroid vector obtained at K = 20, c = 18 indicate the phase relationships among the N = 90 brain areas with respect to the eigenvector. In more detail, all brain areas with positive values are aligned together in phase. (**b**) Brain maps highlight the visual cortex (calcarine, cuneus, lingual, and fusiform) with concurrent frontoparietal, temporal, and hippocampal engagement. (**c**) Increased occupancy of the occipito–parieto–temporal state K20C18 in MDD. Boxplots compare FO between groups (MDD 0.028; HC 0.022; * *p* = 6.17 × 10^−6^; Hedges’ *g* = 0.212). Boxes denote IQR; medians are horizontal lines; whiskers indicate variability; points represent outliers among participants in each group. Summary statistics (median and interquartile range) are shown to facilitate distributional interpretation.

K20C18 engaged the visual cortex (calcarine, cuneus, lingual, and fusiform) with concurrent frontoparietal regions (superior/middle frontal, inferior frontal opercular; superior parietal/postcentral), temporal cortex (middle/inferior temporal), and hippocampal areas (Figure 5).

Patients with MDD showed higher FO than HC (0.028 vs. 0.022), an approximately 27% increase. The between-group difference was statistically significant (*p* = 6.17 × 10^−6^), with a small-to-moderate effect size (Hedges’ *g* = 0.212; 95% CI [0.20, 0.64]) (Figure 5). The spatial profile primarily aligns with the Visual network while integrating control and mnemonic regions, suggesting altered integration or compensatory dynamics in MDD.

### 3.6. Global Redistribution of Temporal Dynamics

**Figure 6:**
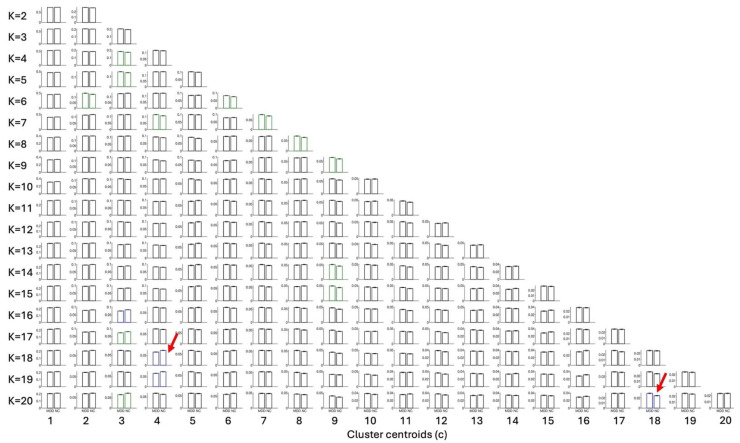
Fractional Occupancy (FO) differences by the phase-locking (PL) state across all 209 states (K = 2 to 20). Each subplot shows the mean FO for MDD and HC. The grid layout has 19 rows (from bottom to top: K = 2 to K = 20) and 20 columns (state index within each K; cells are left blank where the number of states is < 20). Significance after multiple-comparison correction is color coded (blue: *p* < 0.05/209; green: *p* < 0.05/K). Two key findings are highlighted with a red arrow, reduced FO in a state overlapping the posterior DMN (K18C4) in MDD vs. HC, and increased FO in an occipito–parieto–temporal state (K20C18) in MDD vs. HC.

FO differences across LEiDA states were small and bidirectional, indicating redistribution rather than a uniform shift. Patients with MDD showed reduced FO in a posterior DMN state (K18C4) and increased FO in an occipito–parieto–temporal state (K20C18). Signed FO differences (MDD − HC) are shown for all states, with multiple-comparisons–corrected significance highlighted in blue (*p < 0.05/209*) and green (*p* < 0.05/K) the two focal states are indicated by red arrows (Figure 6).

## 4. Discussion

This secondary analysis of the REST-meta-MDD dataset identifies selective, bidirectional alterations in time-resolved network organization in MDD: significantly reduced FO of a posterior DMN state, alongside increased FO in an occipito–parieto–temporal network state, relative to HC [15]. These findings robustly reinforce and extend prior observations from the REST-meta-MDD consortium and other international cohorts, confirming LEiDA’s sensitivity to clinically relevant network dynamics in MDD [7,10,16,24,25].

The decline in FO of the overlapping posterior DMN state, combined with shorter dwell times and elevated switching, highlights fundamental temporal instability within this canonical network in MDD [7,8,22]. Anatomically localized to the posterior cingulate cortex, angular gyrus, and hippocampal–parahippocampal regions—midline territories adjacent to ventricular and perivascular corridors where infra-slow vasomotor and CSF oscillations are prominent; these disruptions target key substrates of self-referential and autobiographical processing [22]. The current results align consistently with an extensive literature documenting DMN hypoconnectivity across static and dynamic paradigms in MDD and reinforce the mechanistic model wherein impaired DMN stability underpins core clinical features such as rumination and emotional dysregulation [7,8,25].

Concurrently, increased FO in the occipito–parieto–temporal network suggests compensatory recruitment of perceptual and executive control systems [10,16]. This shift toward externally oriented, task-positive configurations reflects dynamic network rebalancing in response to DMN disruption, consistent with prior dynamic connectivity studies in MDD [10,16].

The present results, as well as the REST-meta-MDD results, report robust DMN alterations using harmonised pipelines that likely suppress physiology-linked variance in large samples [7]. Persistence of posterior DMN reductions suggests that residual physiology–network coupling survives conservative denoising and topological shifts, motivating longitudinal, hypothesis-driven work [7,24]. The robustness of DMN and occipito–parieto–temporal Fractional Occupancy signatures, despite clinical heterogeneity—60% medicated patients, Hamilton Depression Rating Scale score = 22.7 (SD = 5.4), 52% first-episode—across 848 MDD versus 794 HC participants (17 sites) highlights residual neurophysiological variance surviving denoising and pharmacotherapy [7]. DMN hubs overlie ventricular/perivascular corridors coupling infra-slow BOLD signals [26]. Current monoaminergic agents leave this dynamic intact. Leading Eigenvector Dynamics Analysis-guided multimodal studies in drug-naïve cohorts, targeting pulsatility/glymphatic function, could dissect causal roles, advancing personalized stratification. Notably, REST-meta-MDD DIRECT’s last stage expands beyond predominantly Chinese cohorts to examine ethnicity and adopts a surface-based preprocessing pipeline that improves cross-site comparability [27]. This evolution supports the recommendation to treat preprocessing as a design factor, adopt physiology-aware modelling, and evaluate transportability across ancestry, site, and scanner using harmonisation that preserves diagnostic variance [28].

Despite LEiDA’s robust detection of DMN-linked dynamic disruptions, fundamental mechanistic questions persist [24]. While the dominant hypothesis attributes these fluctuations to synchronized neuronal firing modulated by neurovascular coupling, emerging evidence implicates contributory roles for ultra-slow oscillations, anatomical resonance, and brain fluid dynamics [26,27,28].

In this context, recent findings delineate a compelling network-specific phase alignment between DMN regions—notably the medial prefrontal cortex, posterior cingulate, and angular gyrus—and ventricular signals within defined “BraVe mode II” coupling modes [26]. This provides novel empirical indications of functional interactions between large-scale brain networks and brain fluid dynamics, advancing an integrated neurofluidic–neurophysiological framework for understanding DMN fluctuations, yet demands further empirical validation [26]. While antidepressants modulate static RSN, dynamic FO residuals imply complementary neurofluidic roles [7,26]. Multimodal studies incorporating repeated-measures TMS and ECT—interventions known to modulate dFC—offer promising avenues for causal hypothesis testing [16,29] as does advanced focused ultrasound neuromodulation coupled with concurrent fMRI [28].

Methodological constraints challenge the interpretability and robustness of dFC in BOLD fMRI. BOLD signals are affected by both neural and non-neural physiological confounds, such as vascular dynamics and respiration, which manifest as phase-locking patterns in fMRI data [11]. Discriminating these components is necessary to avoid misinterpretation of dynamic brain states [26].

dFC metrics are highly sensitive to analytic choices, including parcellation size, phase extraction, clustering algorithms, frequency filtering, and temporal windowing. Triangulation across methods with preregistered criteria is essential for reproducibility [10,17,25]. Although LEiDA can detect recurrent, anatomically distinct dynamic modes reflecting both neural and physiological components, this does not fully mitigate analytic variability or physiological confounds, underscoring the need for multimodal data acquisition and robust analytical triangulation [26].

A further limitation is that the current LEiDA analysis was restricted to group-level comparisons between MDD and HC and was not optimized for individual-level diagnostic classification or prognosis. Developing clinically useful biomarkers would require explicitly predictive modelling with cross-validated training and test sets, harmonisation of site-related variance, and integration of longitudinal and treatment data, which the REST-meta-MDD dataset does not yet provide in a uniform way. Nonetheless, the specific pattern of decreased posterior DMN occupancy and increased occipito–parieto–temporal occupancy identified here offers a mechanistically grounded feature space that can be leveraged by future work targeting subject-level prediction in MDD.

Clinical heterogeneity and the predominance of cross-sectional designs in MDD limit insight into temporal network dynamics and treatment effects. Longitudinal studies with detailed symptom profiling, stratified by medication and episode status, and ethnically diverse validation are necessary to develop generalizable biomarkers [7,29]. It remains pertinent, in the context of methodological limitations, that phase coupling detects synchrony but not causality, limiting mechanistic interpretation [26]. Technical and economic challenges associated with multimodal neuroimaging integrating neural, vascular, and fluid dynamics constrain clinical scalability, necessitating rigorous, multimodal, longitudinal frameworks to develop clinically meaningful fMRI biomarkers in MDD [27].

A key limitation is that clinical heterogeneity within MDD (e.g., episode status, illness duration, and symptom severity) was not explicitly modelled, despite partial availability across cohorts. Consequently, the findings reflect average diagnostic-group effects rather than subtype-specific signatures, and the generalisability across MDD subgroups (e.g., first-episode vs. recurrent) remains to be established. Future stratified analyses within REST-meta-MDD and DIRECT will be essential to test whether the observed DMN and occipito–parieto–temporal alterations generalise across, or are driven by, particular clinical profiles.

Another limitation relates to the multicentre nature of REST-meta-MDD: although harmonised protocols and stringent QC were applied, site and scanner effects were not explicitly modelled or stratified. Residual batch effects and field-strength differences (1.5T vs. 3T) may therefore contribute to LEiDA state variance and slightly bias effect sizes, and the robustness of findings across individual sites cannot be formally quantified here. Site-aware harmonisation and stratified validation in future multicentre studies should be required to confirm transportability.

## 5. Conclusions

The present study reinforces LEiDA’s utility in characterizing the temporal reorganization of RSN in MDD and contributes significantly to the evolving understanding of the DMN’s mechanistic underpinnings. This underscores the critical need for integrative neurofluidic–neurophysiological frameworks, marking an essential advancement toward biologically informed, personalized interventions that move beyond conventional symptom-focused treatment paradigms in MDD.

## Data Availability

REST-meta-MDD consortium data available for download at https://rfmri.org/REST-meta-MDD (accessed on 21 July 2025).

## Abbreviations

The following abbreviations are used in this manuscript:

AAL	Automated Anatomical Labeling
BOLD	Blood Oxygenation Level–Dependent
BPS	British Psychological Society
CSF	Cerebrospinal Fluid
dFC	Dynamic Functional Connectivity
DMN	Default Mode Network
DPARSF	Data Processing Assistant for Resting-State fMRI
DSM	Diagnostic and Statistical Manual of Mental Disorders
ECT	Electroconvulsive Therapy
EPI	Echo-Planar Imaging
FO	Fractional Occupancy
FWE	Familywise Error
fMRI	Functional Magnetic Resonance Imaging
FWH	Full Width at Half Maximum
HC	Healthy Controls
HDRS-17	17-Item Hamilton Depression Rating Scale
ICD	International Classification of Diseases
ICMJE	International Committee of Medical Journal Editors
LEiDA	Leading Eigenvector Dynamics Analysis
MDD	Major Depressive Disorder
MNI	Montreal Neurological Institute (Space/Template)
MRC	Medical Research Council
MRI	Magnetic Resonance Imaging
PL	Phase-Locking
QC	Quality Control
REST-meta-MDD	Resting-State fMRI Meta-Analysis of Major Depressive Disorder consortium
RSN	Resting-State Network(s)
rs-fMRI	Resting-State Functional Magnetic Resonance Imaging
SD	Standard Deviation
TE	Echo Time
TMS	Transcranial Magnetic Stimulation
TR	Repetition Time
